# Identification of Essential Proteins Based on Improved HITS Algorithm

**DOI:** 10.3390/genes10020177

**Published:** 2019-02-25

**Authors:** Xiujuan Lei, Siguo Wang, Fangxiang Wu

**Affiliations:** 1 School of Computer Science, Shaanxi Normal University, Xi’an 710119, China; wangsiguo@snnu.edu.cn; 2 Department of Mechanical Engineering and Division of Biomedical Engineering, University of Saskatchewan, Saskatoon, SK S7N 5A9, Canada

**Keywords:** essential proteins, HSEP, HITS algorithm, weighted PPI networks

## Abstract

Essential proteins are critical to the development and survival of cells. Identifying and analyzing essential proteins is vital to understand the molecular mechanisms of living cells and design new drugs. With the development of high-throughput technologies, many protein–protein interaction (PPI) data are available, which facilitates the studies of essential proteins at the network level. Up to now, although various computational methods have been proposed, the prediction precision still needs to be improved. In this paper, we propose a novel method by applying Hyperlink-Induced Topic Search (HITS) on weighted PPI networks to detect essential proteins, named HSEP. First, an original undirected PPI network is transformed into a bidirectional PPI network. Then, both biological information and network topological characteristics are taken into account to weighted PPI networks. Pieces of biological information include gene expression data, Gene Ontology (GO) annotation and subcellular localization. The edge clustering coefficient is represented as network topological characteristics to measure the closeness of two connected nodes. We conducted experiments on two species, namely *Saccharomyces cerevisiae* and *Drosophila melanogaster*, and the experimental results show that HSEP outperformed some state-of-the-art essential proteins detection techniques.

## 1. Introduction

It is well known that proteins are important for living organisms and are the main components of cellular physiological metabolic pathways. Proteins are involved in various biological processes and carry out almost all cellular functions by interacting with other proteins or DNA. With the development of proteomics in the post-genomic era, several protein-related topics have become the major subject of many studies, including the discovery of protein structures and functions, the identification of essential proteins or protein complexes and functional modules. Notably, removing only one of these essential proteins will cause fatal defects on the organism [1]. In addition, recent studies have shown that essential proteins are related to human disease genes and play significant roles in predicting drug targets [2,3]. Therefore, it is important to identify essential proteins, which will help us to understand the minimum requirements of cell life and find new ways to treat diseases.

To date, much work has been done for predicting essential proteins by biological experiment-based methods and network-based essential proteins discovery methods. Although the tradition experimental methods, such as gene knockouts [4], RNA interference [5] and conditional knockouts [6], can provide an accurate prediction of essential proteins, they are time-consuming and expensive. With the development of high-throughput technologies, such as yeast two-hybrid system [7], mass spectrometry analysis [8], snf tandem affinity purification [9] various protein–protein interaction (PPI) data are available. To break through these experimental constraints, some researchers have proposed various computational approaches based on available PPI data. Some studies show that highly-connected proteins in PPI networks tend to be essential ones, which is called the centrality–lethality rule [10]. The absence of highly connected protein nodes in the PPI networks may lead to the collapse of the entire network structure and have a fatal effect on the organism itself. Various network centrality metrics have emerged, such as Degree Centrality (DC) [10], Betweenness Centrality (BC) [11], Closeness Centrality (CC) [12], Subgraph Centrality (SC) [13], Eigenvector Centrality (EC) [14], Information Centrality (IC) [15], Neighborhood Centrality (NC) [16] and Local Average Connectivity (LAC) [17]. Inspired by these studies results, some centrality metrics are used to identify essential proteins; to some extent, they have certain deficiencies due to a high proportion of false positive and false negative in PPI data. Therefore, many methods have been proposed for identifying essential proteins.

Taking into account the shortcomings of the PPI networks, some researchers began to weigh PPI networks by integrating other biological data, including gene expression data, protein complex information, subcellular localization information, orthologous protein information and so on. Li et al. and Peng et al. proposed two methods for identifying essential proteins by combining PPI networks and gene expression data, named PeC [18] and WDC [19], respectively. Some studies indicate that essential proteins are more likely to gather in protein complexes [20]. Based on this point of view, two methods named UC and modified UC-P that integrate protein complex information were proposed by Li et al. [21] to identify essential proteins. Moreover, recently, many studies find that the subcellular localization of proteins may play an important role in identifying essential proteins. Tang et al. proposed a method named CNC that integrates subcellular localization information to improve the precision of detecting essential proteins [22]. Because most essential proteins are conservative, some methods that combine proteins orthology information are proposed, such as SON presented by Li et al. [23]. Meanwhile, some researchers detected essential proteins based on weighted PPI networks. Xu et al. proposed a method named essentiality ranking that integrates multiple data sources to weighted PPI networks [24]. Recently, Peng et al. proposed a new prediction method, named UDoNC, by combining the domain features of proteins with their topological properties in PPI networks [25].

Hypertext induced topic search (HITS) is a famous algorithm in web structure mining, and it was proposed by Kleinberg in 1998 [26]. Kleinberg divided network pages into authority pages and hub pages and then joined them together in the link structure. The former provides best information related to search topics; the more it is cited by network pages, the higher is its authority value. The latter provides important hyperlinks; the more it cites authoritative pages, the higher is its hub value. HITS algorithm is widely applied to web searches, and successfully solves some practical problems, such as web community [27].

In this paper, we present a new computational method with HITS algorithm on weighted PPI networks to identify essential proteins, named HSEP. First, we turn the original undirected PPI network into a directed network. Then, we combine biological information and network topological features to weighted PPI networks and analyze three aspects: false positive and false negative, protein functions and protein positions. Biological data used in this method include gene expression data, Gene Ontology (GO) annotation and subcellular localization data. As a representative of the topological characteristics of the PPI networks, we use the Edge Clustering Coefficient (ECC) to measure the reliability of two connected proteins. Next, we apply the HITS algorithm to the weighted PPI network. Following that, we rank the proteins according to the authority and hub values obtained by the HITS algorithm. Furthermore, we propose an ensemble method to adjust the parameter in HSEP. To validate the proposed method HSEP, we compared HSEP with various existing methods, including DC, EC, IC, SC, NC, LAC, WDC, PeC and UDoNC. All experiments were conducted on the *Saccharomyces cerevisiae* PPI data and *Drosophila melanogaster* data. Experimental results show that our method outperformed the other existing methods.

## 2. Methods

### 2.1. Hypertext Induced Topic Search Algorithm

Hypertext Induced Topic Search (HITS) algorithm was originally proposed to analyze the importance of web pages and is an iterative algorithm. HITS is a search query dependent algorithm that ranks the web page by processing its entire in-links and out-links. In the HITS algorithm, each page is given two attributes: the hub and the authority. The definition is as follows:

**Definition** **1.** ***Authority.** A high quality authority page will be pointed to by many high quality hub pages. The value of the page hub is equal to the sum of the authority values of all the pages it points to*.

**Definition** **2.** ***Hub.** A high quality hub page points to many high quality authority pages. The page authority value is the sum of all the hub values that point to it*.

**Figure 1:**
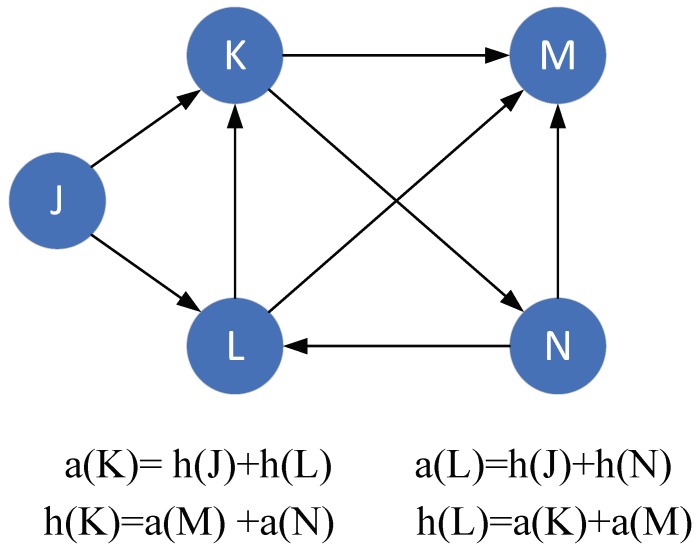
A simple example of calculating hub and authority values.

An example of calculating the value of the hub and authority is shown in Figure 1.

Let a(p) and h(p) represent the authority and hub scores of page *p*, respectively. B(p) and F(p) denote the set of referrer and reference pages of page *p*, respectively. HITS algorithm can be divided into several steps:

(1) Compute a(p) and h(p) in a mutually reinforcing way as follows:

(1) $$a\left(p\right)=\sum_{q\in B(p)}h\left(q\right)$$

(2) $$h\left(p\right)=\sum_{q\in F(p)}a\left(q\right)$$

(2) Divide the authority of all web pages by the highest authority to normalize it:

(3) $$a\left(p\right)=\frac{a(p)}{max(a(p))}$$

Divide the hub of all web pages by the highest hub to normalize it:

(4) $$h\left(p\right)=\frac{h(p)}{max(h(p))}$$

(3) Repeat Step 2 until the difference between the weight in the previous iteration and the weight in the current iteration is less than the set thresholdl the system has entered a stable state and a(u) and h(v) convergence.

### 2.2. Constructing Weighted Protein-Protein Interaction Network

**Figure 2:**
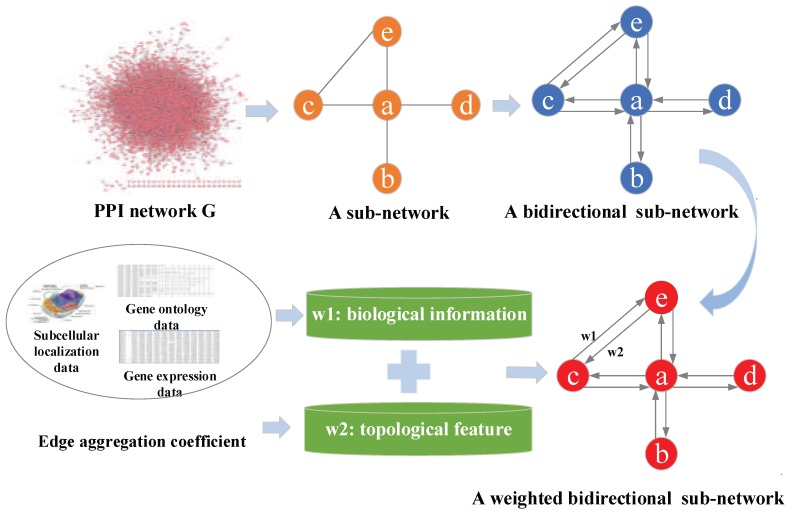
An illustration of weighted PPI network construction.

A protein-protein interaction network usually can be expressed as an undirected graph G=(V,E), where the set of vertices *V* represents proteins, and *E* represents all of interactions between pairs of proteins. To break up the traditional ideas, we assume that the protein interactions are interacting and convert undirected PPI network G=(V,E) into bidirectional network $G^{\prime }=\left(V,E^{\prime }\right)$ that is equivalent to it. It is worth noting that the transformation from undirected graph to directed graph is a mathematical process, which is not applicable to all biological networks, such as the kinase networks. As there are many false positives and false negatives in high-throughput PPI networks, the prediction accuracy will be affected. To solve this situation, we use the biological information and network topological features to weigh edges separately. According to the HITS algorithm, we assume that nodes with high-quality biological information will be pointed by high-quality topological nodes, and high-quality topological nodes will point to high-quality biological information nodes. In Figure 2, an example is shown to explain the weighted PPI network construction.

**Network topology weighted edge.** In general, Edge Clustering Coefficient (ECC) is usually used to evaluate the tightness of two connected proteins. ECC(u,v) can be defined as follows [28]:

(5) $$ECC\left(u,v\right)=\frac{|N_{u}\cap N_{v}|+1}{min\{d_{u},d_{v}\}}$$

where $N_{u}$ and $N_{v}$ denote the set of all neighbors of proteins *u* and *v*, respectively; and $d_{u}$ and $d_{v}$ denote the degree of proteins *u* and *v*, respectively. The weight from node *u* to node *v* is the topological feature ECC.

**Biological information weighted edge.** Gene expression is the process by which information from a gene is used in the synthesis of a functional gene product. Here, we utilize Pearson Correlation Coefficient (PCC), derived from gene expression data, to calculate the importance of related proteins. For gene expression profiles g(u,i)={g(u,1),g(u,2),…,g(u,T)} of protein *u* and g(v,i)={g(v,1),g(v,2),…,g(v,T)} of protein *v*, the PCC is defined as follows:

(6) $$PCC\left(u,v\right)=\sum_{i=1}^{T}\frac{g\left(u,i\right)-\bar{g}\left(u\right)}{\sqrt{{\left(g\left(u,i\right)-\bar{g}\left(u\right)\right)}^{2}}}\cdot \frac{g\left(v,i\right)-\bar{g}\left(v\right)}{\sqrt{{\left(g\left(v,i\right)-\bar{g}\left(v\right)\right)}^{2}}}$$

where $\bar{g}\left(u\right)$ and $\bar{g}\left(v\right)$ represent the average gene expression value of profiles *u* and *v*, respectively. Next, from the perspective of protein functional similarity, whether there are some common GO annotations between two interacting proteins, the two proteins have the same function and the interaction between proteins becomes strong are analyzed. GO [29] is widely used to represent genes and gene products that span different species. To evaluate the semantic similarity between the GO terms to protein annotations in a PPI network, we adopt the method introduced by Wang et al. [30]: the higher is the value, the stronger is the interaction between proteins:

(7) $$GO\_sim\left(u,v\right)=\frac{\sum_{t\in T_{u}⋂T_{v}}\left(S_{u}\left(t\right)+S_{v}\left(t\right)\right)}{\sum_{t\in T_{u}}S_{u}\left(t\right)+\sum_{t\in T_{v}}S_{v}\left(t\right)}$$

where $T_{u}$ and $T_{v}$ are the annotations of proteins *u* and *v*, respectively; $S_{u}\left(t\right)$ is the S-value of GO term *t* related to term *u*; and $S_{v}\left(t\right)$ is the S-value of GO term *t* related to term *v*. For most eukaryotes, subcellular compartments produce specific environments that regulate protein biological processes within cells. Subcellular location is divided into 11 different compartments: cytoskeleton, Golgi apparatus, cytosol, endosome, mitochondrion, plasma membrane, nucleus, extracellular space, vacuole, endoplasmic reticulum, and peroxisome. Some studies have shown that, if proteins with two interacting edges are in the same position, the interaction between proteins becomes more reliable [31]. Therefore, we define SL(u,v) as follows to evaluate the connected proteins by subcellular location information:

(8) $$SL\left(u,v\right)=\frac{C}{C_{max}}$$

where *C* denotes the times of edge (u,v) appears in subcellular location, and $C_{max}$ denotes the max times of edge (u,v) appears in subcellular location. The weight from node *v* to node *u* is the combination of biological information including PCC, GO_sim(u,v) and SL, which is defined as follows:

(9) $$w_{vu}=PCC\left(v,u\right)+GO\_sim\left(v,u\right)+SL\left(v,u\right)$$

### 2.3. Identifying Essential Proteins Based on HSEP Algorithm

Our proposed new algorithm HSEP adopts HITS algorithm based on weighted PPI networks that are constructed in Section 2.2. According to the iteration of the HITS algorithm on the weighted networks, we can obtain the authority value to represent biological information and the hub value to represent topological feature of each protein. To comprehensively evaluate the importance of each protein, we combine the authority value and the hub value to acquire the final score, which can be defined as follows:

(10) HSEP(v)=α×a(v)+(1−α)×h(v)

where α∈[0,1] is used to adjust the proportion of these two scores. If the value of α is equal to 0, the sorting score only depends on the topological information. If the value of α is between 0 and 1, the sorting score is computed based on the biological information and topological feature. According to the definition of HSEP(v), we expect its performance to be affected by different parameters α. To facilitate the application of HSEP to different organisms to identify the essential proteins and minimize the selection pressure of the parameter α, we adopt an ensemble method introduced by Zhang et al. [32]. For each α∈[0,1] (*i* = 1,2, …, *k*), we can get an $HSEP_{i}\left(v\right)$ for each protein *v* and its corresponding rank. According to the score of HSEP, we can obtain *k* ranks of each protein with different *k* values of α. Based on each ranking $HSEP_{i}\left(v\right)$, we select the top *n* ranked proteins, denoted as $X_{i}$, and combine them as the total candidates set *X*. Then, we use ensemble method and majority voting strategy to further predict essential proteins from *X*. Let EM denote the number of times of protein *v* appears in the *X*. If the EM of protein *v* is greater than the threshold *T*($\left\lfloor \frac{k}{2}\right\rfloor$), then the protein *v* is considered to be an essential protein. The EM is defined as follows:

(11) $$EM\left(v\right)=\sum_{i=1}^{k}z\left(v,i\right)$$

(12) $$\begin{array}{c} \left\{\begin{array}{c} z\left(v,i\right)=1,\left(i\in X_{i}\right)\\ z\left(v,i\right)=0,\left(i\notin X_{i}\right) \end{array}\right. \end{array}$$

**Pseudocode of HSEP**

The pseudocode of HSEP algorithm is divided into two steps, as shown in Algorithm 1. The first step weighs PPI networks with gene expression data, GO annotation, subcellular localization data, and topological feature with edge clustering coefficient. The second step applies HITS algorithm on weighted PPI networks.

**Algorithm 1** HSEP essential proteins identification.**Require:** A PPI network G=(V,E), Gene expression data, Subcellular location data Gene Ontology GO. **Ensure:** Essential protein set.     **Step 1**
1:Convert *G* to Bidirectional Digraph $G^{\prime }\left(V,E^{\prime }\right)$2:**for** each interacting protein pair (a,b) in PPI **do**3:  Calculate ECC /*The closeness of the two nodes*/4:  Calculate PCC /*the importance of two nodes based on Gene expression */5:  Calculate GO_sim /*The functional similarity of the two nodes based on GO annotation*/6:  Calculate SL /*the reliable of two nodes based on subcellular localization */7:**end for**8:**for** each interacting protein pair (a,b) in $G^{\prime }$ **do**9:  edge(a,b) = ECC(a,b)10:  edge(b,a) = PCC(b,a)+GO_sim(b,a)+SL(b,a)11:**end for**  **Step 2**12:**for***m* in [1,maxiter] **do**13:  **for** each node *v* in *V* **do**14:    $a_{m}\left(v\right)=\sum_{(u,v)\in E}h_{m-1}\left(u\right)$15:    $h_{m}\left(v\right)=\sum_{(u,v)\in E}a_{m-1}\left(u\right)$16:    $a_{m}=\frac{a_{m}}{max(a_{m})}$17:    $a_{m}=\frac{h_{m}}{max(h_{m})}$18:    m=m+119:    until$|a_{m}-a_{m-1}\left|+\right|h_{m}-h_{m-1}|<\gamma$20:    return $\left(a_{m},h_{m}\right)$21:  **end for**22:**end for**23:calculate ensemble score EM24:a essential proteins set=EM>T

## 3. Results and Discussion

To verify whether our proposed method HSEP is effective for identifying essential proteins, we performed experiments based on *Saccharomyces cerevisiae* data and *Drosophila melanogaster* data, and analyzed the influence of parameter on the experiment results. To demonstrate the performance of HSEP, we compared HSEP with a number of existing methods, including DC, EC, IC, SC, NC, LAC, WDC, PeC and UDoNC. Meanwhile, to further evaluate the performance of HSEP, we used some statistical strategies to compare with other methods. In addition, precision–recall curves were used to analyze the influence of different parameter α on the experimental results. Finally, we analyzed the identified essential proteins to further estimate our proposed method HSEP.

### 3.1. Experimental Data

To demonstrate the effectiveness of our proposed method, we performed experiments based on two species: *Saccharomyces cerevisiae* and *Drosophila melanogaster*. The *Saccharomyces cerevisiae* data are widely used for studying essential proteins currently. We applied two sets of *Saccharomyces cerevisiae* PPI network including DIP database [33] and Gavin database [34]. The PPI network of *Drosophila melanogaster* was constructed using the HINT database [35], which is a curated compilation of high-quality PPIs from eight interatomic resources (BioGRID, MINT, iRefWeb, DIP, IntAct, HPRD, MIPS and the PDB). After the repeated interactions and the self-connecting interactions, the detailed information is listed in Table 1. The subcellular localization information of proteins were retrieved from knowledge channel of COMPARTMENTS database [36]. There are 5974 proteins and 238,620 subcellular locations, which could be classified into 11 localizations. The gene expression data of *Saccharomyces cerevisiae* and *Drosophila melanogaster* were downloaded from GEO database with accession numbers GSE3431 [37] and GSE7763 [38], respectively. GO database is one of the most comprehensive ontology databases in bioinformatics. The GO annotation data of *Saccharomyces cerevisiae* obtained from GO Consortium [39] and the *Drosophila melanogaster* GO annotation data were extracted from the COMPARTMENTS database [36]. The list of known essential proteins covers 1285 and 408 essential proteins of *Saccharomyces cerevisiae* and *Drosophila melanogaster*, respectively, that were collected from MIPS [40], SGD [41], DEG [42], and SGDP [1].

### 3.2. Comparison with Other Identification Measures

**Figure 3:**
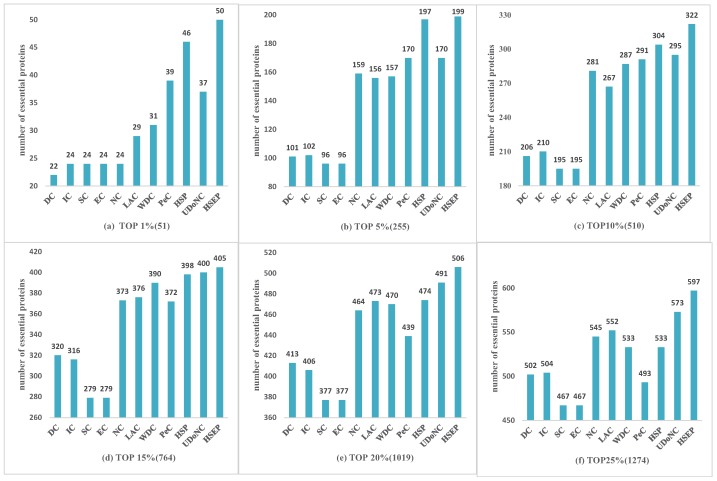
Comparison of HSEP with other essential protein discovery methods: (**a**) Top 1% (Top 51); (**b**) Top 5% (Top 255); (**c**) Top 10% (Top 510); (**d**) Top 15% (Top 764); (**e**) Top 20% (Top 1019); and (**f**) Top 25% (Top 1274).

**Figure 4:**
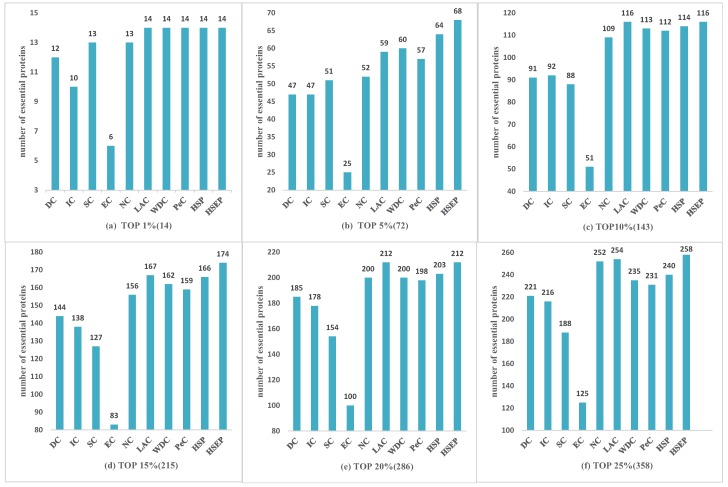
Comparison of HSEP with other essential protein discovery methods on Gavin data. (**a**) TOP 1% (14); (**b**) TOP 5% (72); (**c**) TOP 10% (143); (**d**) TOP 15% (215); (**e**) TOP 20% (286); and (**f**) TOP 25% (358).

**Figure 5:**
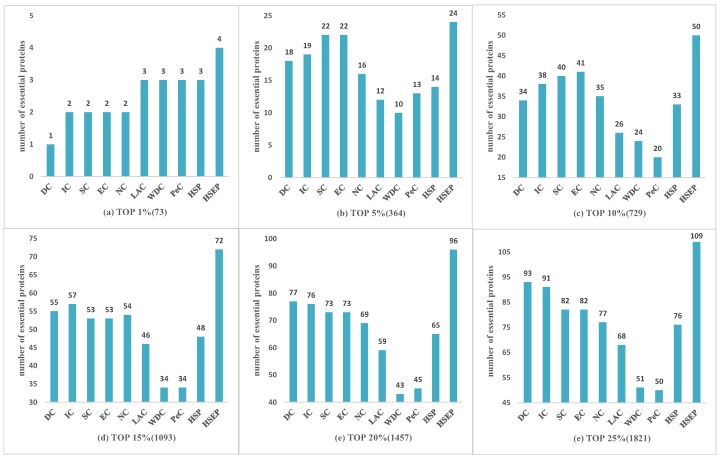
Comparison of HSEP with other essential protein discovery methods on HINT data. (**a**) TOP 1% (73); (**b**) TOP 5% (364); (**c**) TOP 10% (729); (**d**) TOP 15% (1093); (**e**) TOP 20% (1457); and (**f**) TOP 25% (1821).

To evaluate the performance of HSEP, we compared HSEP with other competing methods: DC, EC, IC, SC, NC, LAC, WDC, PeC and UDoNC, and selected the top 1%, 5%, 10%, 15%, 20% and 25% proteins as the candidate set. We set α = (0, 0.1, 0.2, 0.3, 0.4, 0.5, 0.6, 0.7, 0.8, 0.9, 1), and *T* = 5. First, to further demonstrate that the HITS algorithm was effective for identifying essential proteins, in terms of biological information, we only used gene expression data to weigh the protein network, named HSP. Then, the comparison of the prediction results with known essential proteins was expressed in terms of histogram, as shown in Figure 3, Figure 4 and Figure 5, where we can see that the experimental results of HSP are superior to PeC. It indicates that HITS algorithm was effective in identifying essential proteins, since these methods both use gene expression information and ECC to weigh the PPI network. At the same time, HSEP performed better than HSP, which manifests GO annotation and subcellular localization has significant role in identifying essential proteins.

For the DIP dataset shown in Figure 3, our proposed method HSEP clearly performed better than other methods, which indicates that HSEP was effective to identify essential proteins. Especially at the top 1%, 20% and 25%, HSEP method had a more obvious advantage. Taking top 1% (51) as an example, 50 essential proteins were correctly identified by the HSEP while IC, SC and EC correctly predicted 24. At the top 25%, HSEP correctly identified 597 essential proteins, 130 more than SC and EC.

For the Gavin dataset shown in Figure 4, HSEP was slightly better than other eight methods from top 1% to top 25% of ranked proteins. At top 1% (14) level, our proposed method HSEP, LAC and PeC could correctly identify all 14 true essential proteins. The results predicted by HSEP were similar to those obtained using LAC at the top 1%, 10%, 20% and 25% levels. Overall, as shown in Figure 3 and Figure 4, HSEP had more obvious advantages on DIP datasets. Table 1 shows that the density of the Gavin dataset is 3.4 times higher than DIP dataset. We can draw the conclusion that HSEP algorithm was more suitable for dense protein networks on *Saccharomyces cerevisiae*.

For the HINT dataset shown in Figure 5, HSEP exhibited superior performance compared with the other methods from top 1% to 25% of ranked proteins, and it increased the prediction precision by more than 100%, 26%, 31%, 39%, 26%, and 20% at six levels compared with IC. Comparing Figure 5 with Figure 3 and Figure 4, we can see that Figure 5 presents more obvious advantage, demonstrating our proposed method had better performance on *Drosophila melanogaster*.

### 3.3. Validation Using Six Statistical Measures

To further evaluate the performance of our proposed HSEP, we adopted several statistical measures, namely sensitivity (SN), specificity (SP), positive predictive value (PPV), negative predictive value (NPV), F-measure (*F*), and accuracy (ACC), to determine how effectively the essential proteins Were identified by different methods. These statistical measures are defined as follows:

(13) $$SN=\frac{TP}{TP+FN}$$

(14) $$SP=\frac{TN}{TN+FP}$$

(15) $$PPV=\frac{TP}{TP+FP}$$

(16) $$NPV=\frac{TN}{TN+FN}$$

(17) $$F-measure=\frac{2\times SN\times PPV}{SN+PPV}$$

(18) $$ACC=\frac{TP+TN}{TP+TN+FP+FN},$$

where TP is the number of essential proteins correctly identified as essential proteins, FP is the number of nonessential proteins mistakenly identified as essential proteins, TN is the number of nonessential proteins correctly identified as nonessential proteins, and FN is the number of essential proteins mistakenly identified as nonessential proteins. The comparisons of SN, SP, PPV, NPV, F−measure and ACC of HSEP and other methods are shown in Table 2. As shown in Table 2, the HSEP had a better quality than other methods, and we could get similar conclusions with those shown in Figure 3, Figure 4 and Figure 5.

### 3.4. Influence of Parameter α on HSEP Based on Precision–Recall Curves

**Figure 6:**
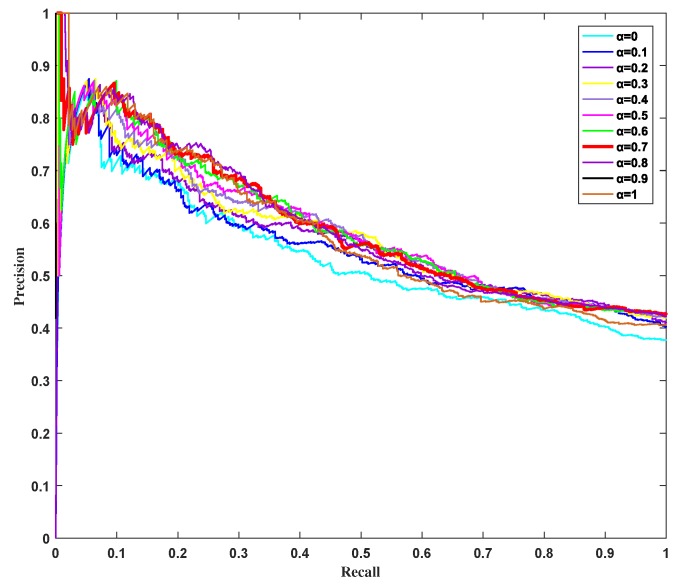
Precision–recall curves of HSEP with different α.

To investigate the influence of parameter α on HSEP, precision–recall curves were used to assess the generality of our method. The precision and recall of the top *n* ranked proteins are defined as follows:

(19) $$Precision\left(n\right)=\frac{TP(n)}{TP(n)+FP(n)}$$

(20) $$Recall\left(n\right)=\frac{TP(n)}{P}$$

where TP(n) is the number of true essential proteins identified correctly, FP(n) is the number of true essential proteins identified incorrectly among the top n proteins, and *P* is the number of true essential proteins in total. Figure 6 shows the PR curves of HSEP with different parameter α on the DIP database. The higher is the curve, the better is the corresponding metric that distinguishes between the essential protein and the non-essential proteins. As shown in Figure 6, the results were the best when α=0.7 and α=0.8. When α=0, namely only biological information was used, the result was worst. Comprehensively, biological information played a more important role than topological properties in identifying essential proteins.

### 3.5. The Analysis of Essential Proteins

**Figure 7:**
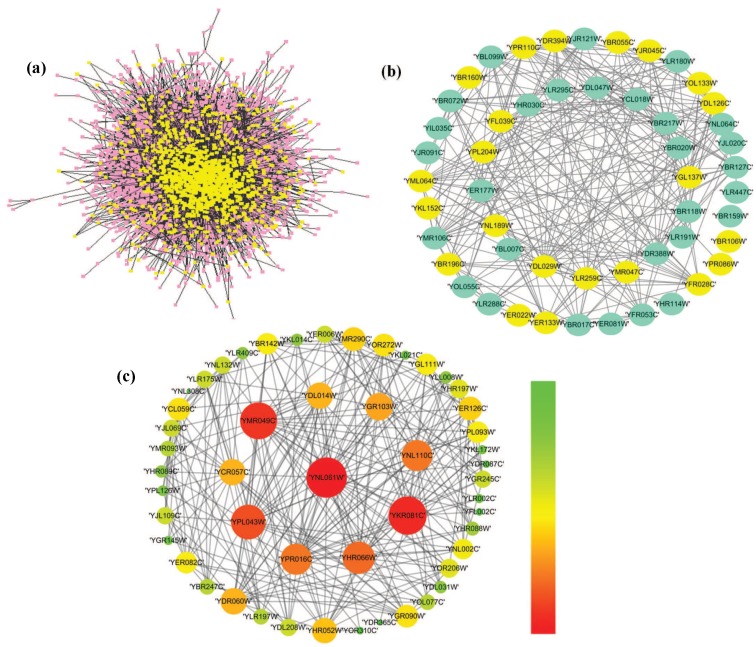
The distribution of essential proteins: (**a**) the identified top 1% essential proteins by DC; (**b**) the identified top 1% essential proteins by DC, where yellow circles are the essential proteins that DC predicted as essential, while aqua circles are the non-essential proteins that DC predicted as essential ones; and (**c**) the identified top 1% essential proteins of HSEP, where the larger is the degree of the protein, the bigger is the size of the protein.The color key indicates that the degree of protein gradually increases from top to bottom.

We analyzed the identified essential proteins on DIP database to further substantiate the performance of our proposed HSEP. Figure 7 shows the overall results in terms of the distribution of known essential proteins in PPI network (Figure 7a), the identified 1% essential proteins by DC (Figure 7b) and the identified 1% essential proteins by HSEP (Figure 7c). In Figure 7, we can see that the number of essential proteins correctly identified by DC was 22, shown as yellow circles. Here, we mainly analyzed the 1% identified essential proteins by HSEP. In Figure 7c, we can see that all top 1% essential proteins are connected to form one subnetwork, which shows good topological features and manifests essential proteins perform biological functions as a module that is of significance for identifying protein complexes. In addition, the protein “YHR066W” has a large degree, but is the only one that wasmistakenly identified as an essential protein, indicating that degree cannot fully reflect the essentiality of proteins.

## 4. Conclusions

Identifying essential proteins is of great importance for understanding the molecular mechanisms of cellular life. In this study, we have presented a new computational method with HITS algorithm on weighted PPI networks to predict essential proteins. Both biological information and network topology are used to weighted PPI networks, which plays an important role in identifying essential proteins. Meanwhile, we apply an ensemble method to avoid the influence of parameter. To investigate the performance of our proposed algorithm, we carried out a group of simulation experiments on the two species of PPI data: *Saccharomyces cerevisiae* and *Drosophila melanogaster*. The experimental results show that HSEP achieved better performance than other methods: DC, EC, IC, SC, NC, LAC, WDC, PeC and UDoNC. To further measure our method, we used six statistical measures to compare with others. In addition, we analyzed the identified essential proteins and they have good topological properties. As future work, our proposed HSEP may be helpful to other studies, such as gene and disease prediction.

## Figures and Tables

**Table 1:** The detail information of the experimental data.

Database	Proteins	Interactions	Density	GO Annotation	Gene Expression	Essential Proteins
DIP	5093	24,743	0.0019	5061	4981	1167
Gavin	1430	6531	0.0064	1430	1418	617
HINT	7285	24,436	0.0009	4878	6999	216

**Table 2:** Comparative analysis of HSEP and the other methods in terms of SN, SP, PPV, NPV, F−measure, and ACC on the PPI networks.

Database	Method	*SN*	*SP*	*PPV*	*NPV*	*F-Measure*	*ACC*
	DC	0.4302	0.8033	0.3940	0.8258	0.4113	0.7178
	IC	0.4319	0.8038	0.3956	0.8263	0.4129	0.7186
	SC	0.4002	0.7944	0.3666	0.8167	0.3826	0.7040
	EC	0.4002	0.7944	0.3666	0.8167	0.3826	0.7040
	NC	0.4670	0.8143	0.4278	0.8371	0.4465	0.7347
DIP	LAC	0.4730	0.8161	0.4333	0.8389	0.4523	0.7374
	WDC	0.4567	0.8112	0.4184	0.8339	0.4367	0.7300
	PeC	0.4225	0.8010	0.3870	0.8235	0.4039	0.7143
	HSP	0.4567	0.8112	0.4184	0.8339	0.4367	0.7300
	UDoNC	0.4910	0.8214	0.4498	0.8444	0.4695	0.7457
	HSEP	**0.5116**	**l0.8275**	**0.4686**	**0.8507**	**0.4891**	**0.7551**
	DC	0.3582	0.8313	0.6173	0.6303	0.4533	0.6270
	IC	0.3501	0.8251	0.6034	0.6256	0.4431	0.6200
	SC	0.3047	0.7906	0.5251	0.5994	0.3856	0.5808
	EC	0.2026	0.7131	0.3492	0.5406	0.2564	0.4927
Gavin	NC	0.4084	0.8695	0.7039	0.6592	0.5169	0.6704
	LAC	0.4117	0.8719	0.7095	0.6611	0.5210	0.6732
	WDC	0.3809	0.8485	0.6564	0.6433	0.4821	0.6466
	PeC	0.3744	0.8103	0.6000	0.6303	0.4611	0.6211
	HSP	0.3890	0.8547	0.674	0.6480	0.4923	0.6536
	HSEP	**0.4182**	**0.8768**	**0.7207**	**0.6648**	**0.5292**	**0.6788**
	DC	0.4306	0.7555	0.0511	0.9775	0.0913	0.7459
	IC	0.4213	0.7552	0.0500	0.9771	0.0893	0.7453
	SC	0.3796	0.7540	0.0450	0.9755	0.0805	0.7429
	EC	0.3796	0.7540	0.0450	0.9755	0.0805	0.7429
HINT	NC	0.3565	0.7533	0.0423	0.9746	0.0756	0.7415
	LAC	0.3148	0.7520	0.0373	0.9729	0.0668	0.7390
	WDC	0.2361	0.7496	0.0280	0.9698	0.0501	0.7343
	PeC	0.2315	0.7494	0.0275	0.9696	0.0491	0.7341
	HSP	0.3519	0.7531	0.0417	0.9744	0.0746	0.7412
	HSEP	**0.5046**	**0.7578**	**0.0599**	**0.9804**	**0.1070**	**0.7503**
